# Nephrolithiasis in sarcoidosis: epidemiology, risk factors, and clinical implications

**DOI:** 10.1007/s00345-025-05923-8

**Published:** 2026-01-30

**Authors:** Giovanni Scala Marchini, Sabrina T. Reis, Filipe A. Correia, Fabio Cesar Miranda Torricelli, Alexandre Danilovic, Fabio Vicentini, Carlos Alfredo Batagello, Ronaldo Adib Kairalla, Alexandre de Melo Kawassaki, Fabio Eiji Arimura, Patrícia Candido, Rodrigo Perrella, William Carlos Nahas, Eduardo Mazzucchi

**Affiliations:** 1 Divisão de Urologia, Hospital das Clínicas HCFMUSP, Faculdade de Medicina, Universidade de São Paulo, Av. Dr. Enéas Carvalho de Aguiar, 255, 7º andar, Cerqueira César, São Paulo, SP 05403-000 Brazil; 2 Laboratório de Investigação Médica 55 (LIM55), Faculdade de Medicina, Hospital das Clínicas HCFMUSP, Universidade de São Paulo, São Paulo, 01246903 SP Brazil; 3 Moriah Institute of Science and Education (MISE), Hospital Moriah, São Paulo, Brazil; 4 Divisão de Pneumologia, Faculdade de Medicina, Instituto do Coração, InCor, Hospital das Clínicas, Universidade de São Paulo, São Paulo, SP Brasil; 5 Uro-Oncology Group, Urology Department, Institute of Cancer State of São Paulo (ICESP), São Paulo, 01246000 SP Brazil

**Keywords:** Sarcoidosis, Nephrolithiasis, Hypercalcemia, Risk factors, Urology

## Abstract

**Objective:**

To describe the demographic profile and risk factors for kidney stone formation in patients with sarcoidosis.

**Material and methods:**

158 sarcoidosis patients were analyzed, comparing groups with and without kidney stones evaluating clinical and metabolic factors and medication use. Statistical analysis was carried out using R software (*p* < 0.05).

**Results:**

The sample consisted of 138 patients (87.34%), with a majority of females (67.4%) and a median age of 54. Frequent comorbidities included hypertension (38.4%), diabetes (18.1%), and dyslipidemia (6.5%). Nephrolithiasis was reported by 11.9% of patients. Laboratory tests showed hypercalcemia in 9.4% and hypercalciuria in 17.4%. Kidney stones were found in 15.9% of patients, three of whom were bilateral. The comparative analysis revealed a significant association with a previous history of nephrolithiasis (40% vs. 6.6%). There was no statistical correlation with laboratory tests, except for uric acid, which was lower in the group with stones. Hydroxychloroquine was more frequent in the group with stones but without statistical significance. Logistic regression did not identify any significant associations.

**Conclusion:**

Nephrolithiasis occurred in 16% of sarcoidosis patients and was more prevalent in women and adults. Calcium disturbances persist, requiring continuous monitoring. A history of renal lithiasis should be valued in diagnosis and follow-up.

## Introduction

Sarcoidosis is a systemic granulomatous disease of unknown etiology, characterized by the formation of non-caseating epithelioid granulomas in various organs [1, 2]. The incidence of sarcoidosis varies globally, being more common in African-Americans (36/100,000) than in Caucasians (11/100,000) [3]. In Europe, the incidence rate is higher in northern countries, such as Sweden (121/100,000), and lower in southern countries, such as Spain (1.36/100,000) [4]. In Brazil, there is no recent epidemiological data, and the latest prevalence estimate is 10 cases per 100,000 inhabitants [5].

Although the lungs are the most frequently affected, kidney involvement can also occur, mainly due to calcium metabolism disorders, resulting in hypercalcemia and hypercalciuria [6]. Studies indicate that hypercalcemia occurs in 10 to 17% of patients, while hypercalciuria is present in up to 62% of cases [7, 8]. The underlying mechanism involves the production of 1alpha-hydroxylase by sarcoid granulomas, promoting the conversion of 25-hydroxyvitamin D into its active form, 1,25-dihydroxyvitamin D (calcitriol), which increases intestinal calcium absorption [9, 10].

Hypercalcemia and hypercalciuria can lead to renal complications such as nephrolithiasis and nephrocalcinosis. Nephrolithiasis occurs in approximately 10% of patients with sarcoidosis and, in some cases, maybe the first sign of the disease [8, 11]. On the other hand, nephrocalcinosis, which is less common, is present in less than 5% of patients and is more common in those with kidney failure [10]. In addition, a renal biopsy can show epithelioid granulomas, characteristic of sarcoidosis, in up to 40% of cases of nephrocalcinosis [8, 12].

Sarcoidosis is not usually considered a urological disease, but it can affect the urinary tract and lead to conditions such as nephrolithiasis. In addition, it can mimic severe urological disorders such as testicular nodules and renal masses, resulting in misdiagnoses of malignancy. The profile of the urological manifestations of sarcoidosis is still not fully understood [13], and no population studies in Brazil evaluate these manifestations or predictors of severity. A follow-up study of patients, analyzing the demographic, metabolic, and radiological profile, could help to understand the risk factors for kidney stone formation and identify prognostic predictors, allowing for more appropriate management of the disease.

This study aimed to describe the epidemiological profile of sarcoidosis patients treated at a public hospital, analyze the prevalence and factors associated with kidney stone formation, correlate the stage of the disease with urological findings, stratify severity, and identify predictive and protective factors for urological metabolic manifestations.

## Materials and methods

### Study design

The evaluation used data from patients seen at the Hospital das Clínicas of the University of São Paulo Medical School (HCFMUSP), registered on the REDCap platform. The patients were divided into two groups according to the presence or absence of kidney stones, and the characteristics of these groups were compared using hypothesis tests. Variables such as age, gender, comorbidities, history of kidney stones, clinical stage of the disease, and symptoms related to sarcoidosis were assessed. Laboratory tests and the use of medication to control sarcoidosis were also analyzed.

The study was approved by the HCFMUSP Ethics Committee for Research Project Analysis (CAPPesq) (number 2.758.607) and included patients being followed up at the HCFMUSP Pulmonology Department over three years. The patients were evaluated in a dedicated urological consultation, with prospective requests for subsidiary and laboratory tests.

### Patient selection

Patients over 18 with a diagnosis of sarcoidosis who agreed to participate in the study by signing an informed consent form were included. Patients with other pulmonary granulomatous diseases or without a definitive diagnosis of sarcoidosis were excluded, as were those who did not undergo the necessary tests, such as abdominal and pelvic CT scans, serum laboratory tests, and 24-hour urine laboratory analysis.

### Research

The demographic characteristics of the patients were analyzed, including gender, age, race, personal history, comorbidities, weight, height, body mass index (BMI), and the date of the first visit to the Departments of Pulmonology and Urology at HCFMUSP. About the diagnosis of sarcoidosis, data was collected on the clinical stage of the disease, symptoms, date of diagnosis, initial and current chest x-ray, form of diagnosis, lung, lymph node or peripheral biopsy, and bronchoalveolar lavage, as well as analysis of other affected systems.

Medications that influence serum and urinary calcium levels and other substances associated with the formation of nephrolithiasis were checked, including hydrochlorothiazide, allopurinol, potassium citrate, and losartan. The main drugs used to treat sarcoidosis, such as corticoids, chloroquine, azathioprine, methotrexate, and anti-TNF, were also evaluated. Serum analysis included sodium, total calcium, ionizable calcium, creatinine, urea, uric acid, potassium, phosphorus, PTH, vitamin D, venous blood gas, free T4, TGO, TGP, DHL, and ESR. The 24-hour urine was analyzed for total volume, calcium, creatinine, oxalate, citrate, sodium, and uric acid. Type 1 urine was analyzed for pH and crystal characteristics, if present.

A helical computed tomography scan of the whole abdomen without contrast, considered the gold standard for diagnosing kidney stones, was requested to assess the presence of stones, except in cases where the patient had already undergone a similar scan in the three months before the consultation. When present, the stones were analyzed for location, size, and density (described in Hounsfield Units - HU). Surgical procedures performed to treat the pathology were also recorded. All the data was tabulated in a specific spreadsheet for analysis and comparison.

### Statistical analysis

Statistical analysis was carried out using R software. Continuous variables were expressed as mean, median, standard deviation, minimum, and maximum, while categorical variables were expressed as absolute numbers and percentages. Fisher’s exact and Pearson’s chi-square tests were used to compare the qualitative variables, while the Mantel-Haenzel chi-square test was used for the ordinal variables. Quantitative variables were analyzed using the Wilcoxon rank sum and Kruskal-Wallis tests.

Logistic regression models were used to investigate the effect of the compared variables on the chance of kidney stones occurring, considering factors such as 24-hour calcium, parathormone dosage, uric acid, age, gender, hydroxychloroquine use and the disease’s current clinical stage. The level of significance was set at a p-value below 5%. All considered a p-value below 5% to be statistically significant.

## Results

### Demographic and clinical data on sarcoidosis

Between February 2019 and June 2022, 158 patients diagnosed with sarcoidosis were initially included, and their demographic and clinical data were collected. Of the excluded patients, two patients did not return. They missed follow-up appointments during the period, and 18 patients returned for follow-up but could not complete the tests required for the analysis. The final analysis included 138 patients, corresponding to 87.34% of the cases initially included.

Of the 138 patients analyzed, 67.4% were female and 32.6% male (Table 1). The median age was 54 years, with an interquartile range (IQR) of 47 to 60 years. In the initial questionnaire, 15 patients (11.9%) reported a history of nephrolithiasis. The average BMI was 29.4, with an IQR of 27.8 to 32.1. In addition, 38.4% had systemic arterial hypertension, 18.1% had diabetes mellitus, and 6.5% had dyslipidemia. There were no records of patients with previous AMI. Other comorbidities were present in 18.8% of cases.

**Table 1:** Demographic and clinical data and stage of disease of the sample

Features	*N* = 138^a^	No information
Age	54.0 (47.0, 60.0)	0
Sex		0
Female	93 (67.4%)	
Male	45 (32.6%)	
Previous kidney stones	15 (11.9%)	12
Systemic arterial hypertension	53 (38.4%)	0
Diabetes Mellitus	25 (18.1%)	0
Dyslipidemia	9 (6.5%)	0
Previous AMI	0 (0.0%)	0
Other comorbidities	26 (18.8%)	0
Initial clinical stage		21
0	2 (1.7%)	
I	20 (17.1%)	
II	63 (53.8%)	
III	8 (6.8%)	
IV	24 (20.5%)	
Current clinical stage		19
0	2 (1.7%)	
I	19 (16.0%)	
II	65 (54.6%)	
III	9 (7.6%)	
IV	24 (20.2%)	
Symptoms		
Asymptomatic	6 (4.3%)	0
Dyspnea	103 (74.6%)	0
Coughing	87 (63.0%)	0
Change in x-ray	1 (0.7%)	0
Changes in other organs	8 (5.8%)	0
Other symptoms	24 (17.4%)	0
Other systems affected	92 (73.0%)	12
Systems		
Cardiovascular	6 (4.3%)	0
Cutaneous	19 (13.8%)	0
Ocular	12 (8.7%)	0
Others	7 (5.1%)	0
Diagnosis		10
Biopsy	113 (88.3%)	
Clinical	15 (11.7%)	
Biopsy site		
Lungs	56 (40.6%)	0
Chest/Mediastinum lymph nodes	22 (15.9%)	0
Skin	22 (15.9%)	0
Lymph nodes (Other)	16 (11.6%)	0
Kidney	0 (0.0%)	0
Others	8 (5.8%)	0

^a^Median (IIQ); n (%)

Regarding the clinical stage of the disease, two patients (1.7%) were in stage 0, 20 patients (17.1%) in stage I, 63 patients (53.8%) in stage II, eight patients (6.8%) in stage III, 24 patients (20.5%) in stage IV, and 21 patients did not have a chest X-ray for classification. At the final assessment, two patients (1.7%) were in stage 0, 19 patients (16.0%) in stage I, 65 patients (54.6%) in stage II, nine patients (7.6%) in stage III, 24 patients (20.2%) in stage IV, and 19 patients had no data available.

As for respiratory symptoms, the most common were dyspnea (74.6%) and cough (63.0%). Other symptoms accounted for 17.4%, and six patients (4.3%) were asymptomatic. In addition, 73.0% of patients had involvement of systems other than the lungs. The most affected systems were cutaneous (13.8%), ocular (8.7%) and cardiovascular (4.3%). Regarding diagnosis, 88.3% of the initial examinations were retrieved. The most common locations for biopsy were the lung (40.6%), chest/mediastinal lymph nodes (15.9%), and skin (15.9%). Other locations were lymph nodes (11.6%) and other areas (5.8%).

### Laboratory tests

Laboratory tests showed significant changes. Total serum calcium was elevated in 9.4% of patients and ionic calcium in 16.8%. Alterations in Vitamin D metabolism were observed in 85.3% of patients, with reduced values. Parathormone was elevated in 44.4% of patients. About urinary disorders, hypercalciuria was observed in 17.4% of patients. In addition, 73.7% had low urinary volume, and 26.3% had hypocitraturia, which can contribute to kidney stones formation (Table 2).

**Table 2:** Classification of serum and urine laboratory tests

Features	*N* = 138^a^
Total serum calcium	
Normal	123 (89.1%)
High total calcium	13 (9.4%)
Reduced total calcium	2 (1.4%)
Serum ionic calcium	
Normal	108 (82.4%)
High ionic calcium	22 (16.8%)
Reduced ionic calcium	1 (0.8%)
Serum creatinine	
Normal	107 (77.5%)
Elevated creatinine	28 (20.3%)
Low creatinine	3 (2.2%)
Serum uric acid	
Normal	70 (71.4%)
Hyperuricemia	26 (26.5%)
Hypouricemia	2 (2.0%)
Parathormone	
Normal	65 (55.6%)
High	52 (44.4%)
Vitamin D	
Reduced vitamin D	93 (85.3%)
Normal	16 (14.7%)
Calcium 24 h	
Normal	114 (82.6%)
Hypercalciuria	24 (17.4%)
Creatinine 24 h	
Normal	104 (78.8%)
Reduced creatinine	25 (18.9%)
Elevated creatinine	3 (2.3%)
Citrate 24 h	
Normal	73 (73.7%)
Hypocitraturia	26 (26.3%)
Oxalate 24 h	
Normal	84 (94.4%)
Hyperoxaluria	5 (5.6%)
Uric acid 24 h	
Normal	70 (83.3%)
Hyperuricosuria	14 (16.7%)
24 h urine volume	
Urine volume below 2 L	98 (73.7%)
Normal	35 (26.3%)

^a^n (%)

### Use of medication

Table 3 shows the medications used by the patients. Hydrochlorothiazide (18.1%), Losartan (16.7%), allopurinol (2.9%), furosemide (1.4%), and Captopril (1.4%) stood out among the drugs related to protective and promoting factors for kidney stones, No patient used potassium citrate. About the treatment of sarcoidosis, 14.5% of patients used hydroxychloroquine, 38.0% used corticosteroids, 39.4% had previously used corticosteroids, and 22.6% had never used them. Methotrexate was used by 24.3% of patients; 22.8% had used it previously, and 52.9% had never used it. Other less frequent medications included mycophenolate mofetil (1.9% in use and 2.8% previously), cyclophosphamide (0.9% in use and 3.7% previously), and azathioprine (5.3% in use and 2.3% previously).

**Table 3:** Comparative analysis - use of medication

	Presence of kidney stones		
Features	Yes, *N* = 22^a^	No, *N* = 116^a^	p-value^b^
Hydrochlorothiazide	2 (9.1%)	23 (19.8%)	0.366
BRA - Losartan, etc.	3 (13.6%)	20 (17.2%)	> 0.999
Allopurinol	0 (0.0%)	4 (3.4%)	> 0.999
Furosemide	0 (0.0%)	2 (1.7%)	> 0.999
ACEI - Captopril, etc.	0 (0.0%)	2 (1.7%)	> 0.999
Litocit	0 (0.0%)	0 (0.0%)	
Hydroxychloroquine	6 (27.3%)	14 (12.1%)	0.093
Other medications	17 (77.3%)	86 (74.1%)	0.757
Azathioprine			0.098
In use	1 (4.8%)	6 (5.4%)	
Previous use	2 (9.5%)	1 (0.9%)	
Never used	18 (85.7%)	105 (93.8%)	
Corticosteroids			0.380
In use	11 (50.0%)	41 (35.7%)	
Previous use	8 (36.4%)	46 (40.0%)	
Never used	3 (13.6%)	28 (24.3%)	
Methotrexate			0.390
In use	6 (27.3%)	27 (23.7%)	
Previous use	7 (31.8%)	24 (21.1%)	
Never used	9 (40.9%)	63 (55.3%)	

^a^n (%)

^b^Pearson’s chi-square test; Fisher’s exact test

### Location and composition of calculi

On CT scans of the abdomen and pelvis, 22 patients (15.9%) with sarcoidosis had kidney stones, 3 of which were bilateral. In the right renal unit, 15 patients had stones in 19 different locations. Of these, 12 patients had stones smaller than 4 mm, three with 5–8 mm stones, and one patient with 12 mm ureterolithiasis. In the left renal unit, nine patients had stones in 11 different locations, with seven patients having stones up to 4 mm, one patient with a 5 mm stone, and one patient with multiple ureteral stones (6 and 8 mm). Two patients with ureteral calculi underwent surgical intervention, while the others were followed up for specific treatment. Stone composition was available for four patients: three stones consisted of calcium oxalate and one of calcium phosphate.

### Comparative analysis between patients with and without kidney stones

A comparative analysis was carried out between two groups: group 1, made up of patients with kidney stones, and group 2, made up of sarcoidosis patients without kidney stones. In the analysis of demographic and clinical data, a history of nephrolithiasis was identified as a positive factor for the risk and presence of kidney stones. Among the 22 patients with kidney stones, 8 (40%) had a history of nephrolithiasis, compared to 6.6% in patients without kidney stones. Other characteristics, such as gender, age, and previous comorbidities, showed no statistically significant difference between the groups (Table 4).

**Table 4:** Comparative analysis of demographic and clinical data and disease stage in patients with sarcoidosis and nephrolithiasis

	Presence of kidney stones		
Features	Yes, *N* = 22^a^	No, *N* = 116^a^	p-value^b^
Age	53.5 (45.0, 60.0)	54.0 (48.0, 60.2)	0.782
Sex			0.560
Female	16 (72.7%)	77 (66.4%)	
Male	6 (27.3%)	39 (33.6%)	
BMI	27.9 (27.6, 29.7)	30.3 (28.1, 32.2)	0.215
Previous kidney stones	8 (40.0%)	7 (6.6%)	**< 0.001**
Systemic arterial hypertension	7 (31.8%)	46 (39.7%)	0.488
Diabetes Mellitus	3 (13.6%)	22 (19.0%)	0.765
Dyslipidemia	0 (0.0%)	9 (7.8%)	0.354
Previous AMI	0 (0.0%)	0 (0.0%)	
Other comorbidities	2 (9.1%)	24 (20.7%)	0.249
Initial clinical stage			0.221
0	0 (0.0%)	2 (2.0%)	
I	5 (27.8%)	15 (15.2%)	
II	10 (55.6%)	53 (53.5%)	
III	1 (5.6%)	7 (7.1%)	
IV	2 (11.1%)	22 (22.2%)	
Current clinical stage			0.802
0	0 (0.0%)	2 (2.0%)	
I	4 (22.2%)	15 (14.9%)	
II	11 (61.1%)	54 (53.5%)	
III	1 (5.6%)	8 (7.9%)	
IV	2 (11.1%)	22 (21.8%)	
Symptoms			
Asymptomatic	2 (9.1%)	4 (3.4%)	0.244
Dyspnea	15 (68.2%)	88 (75.9%)	0.448
Coughing	15 (68.2%)	72 (62.1%)	0.586
Other symptoms	4 (18.2%)	20 (17.2%)	> 0.999
Other systems affected	15 (75.0%)	77 (72.6%)	0.827
Systems			
Cardiovascular	0 (0.0%)	6 (5.2%)	0.589
Cutaneous	4 (18.2%)	15 (12.9%)	0.506
Ocular	1 (4.5%)	11 (9.5%)	0.690
Others	1 (4.5%)	6 (5.2%)	> 0.999
Biopsy site			
Lungs	8 (36.4%)	48 (41.4%)	0.660
Chest/Mediastinum lymph nodes	3 (13.6%)	19 (16.4%)	> 0.999
Skin	7 (31.8%)	15 (12.9%)	0.050
Lymph nodes (Other)	3 (13.6%)	13 (11.2%)	0.721
Kidney	0 (0.0%)	0 (0.0%)	
Others	2 (9.1%)	6 (5.2%)	0.613

^a^Median (IQR); n (%)

^b^ Wilcoxon rank sum test; Pearson chi-square test; Fisher’s exact test; Mantel-Haenzel chi-square test.

The analysis of patients by clinical stage of the disease, both in the initial assessment and in the current stage, did not identify subgroups with a higher risk of kidney stones. Evaluation of the clinical presentations of sarcoidosis, extrapulmonary involvement, and serum tests revealed no association with the presence of kidney stones, except for uric acid, which showed significantly lower values in group 1. Analysis of 24-hour urine also showed no association with the presence of rocks (Table 5). Investigation of sarcoidosis treatment showed no association with the presence of stones, although Hydroxychloroquine showed a greater tendency in group 1 without statistical significance (Table 6).

**Table 5:** Comparative analysis of laboratory tests

	Presence of kidney stones		
Features	Yes, *N* = 22^a^	No, *N* = 116^a^	p-value^b^
Total serum calcium (mg/dL)	9.6 (9.3, 9.9)	9.6 (9.3, 9.9)	0.963
Parathormone	44.0 (33.0, 59.0)	43.0 (33.0, 58.2)	0.951
Uric acid	4.5 (4.0, 5.2)	5.4 (4.5, 6.8)	**0.038**
Serum creatinine	0.8 (0.7, 1.0)	0.8 (0.8, 1.0)	0.456
Creatinine	87.4 (73.1, 96.0)	88.7 (70.1, 128.1)	0.635
Vitamin D	20.9 (17.5, 26.2)	21.9 (16.2, 27.8)	0.722
24-hour urine test			
Urine volume	1,550.0 (1,156.2, 1,957.5)	1,700.0 (1,150.0, 2,100.0)	0.485
Calcium	170.7 (118.1, 243.7)	147.2 (77.5, 203.4)	0.152
Oxalate	19.0 (13.0, 22.0)	19.5 (11.2, 26.8)	0.805
Citrate	495.0 (310.0, 640.0)	447.0 (317.8, 672.5)	0.978
Uric acid	0.4 (0.3, 0.5)	0.5 (0.3, 0.6)	0.829
Adjusted calciuria	212.7 (132.0, 285.0)	175.8 (91.9, 247.6)	0.167

^a^Median (IIQ).

^b^Wilcoxon rank sum test; Pearson chi-square test; Fisher’s exact test; Mantel-Haenzel chi-square test

**Table 6:** Comparative analysis - use of medication

	Presence of kidney stones		
Features	Yes, *N* = 22^a^	No, *N* = 116^a^	p-value^b^
Corticosteroids			0.380
In use	11 (50.0%)	41 (35.7%)	
Previous use	8 (36.4%)	46 (40.0%)	
Never used	3 (13.6%)	28 (24.3%)	
Methotrexate			0.390
In use	6 (27.3%)	27 (23.7%)	
Previous use	7 (31.8%)	24 (21.1%)	
Never used	9 (40.9%)	63 (55.3%)	
Azathioprine			0.098
In use	1 (4.8%)	6 (5.4%)	
Previous use	2 (9.5%)	1 (0.9%)	
Never used	18 (85.7%)	105 (93.8%)	
Hydroxychloroquine	6 (27.3%)	14 (12.1%)	0.093
BRA - Losartan, etc.	3 (13.6%)	20 (17.2%)	> 0.999
Hydrochlorothiazide	2 (9.1%)	23 (19.8%)	0.366
Allopurinol	0 (0.0%)	4 (3.4%)	> 0.999
Furosemide	0 (0.0%)	2 (1.7%)	> 0.999
ACEI - Captopril, etc.	0 (0.0%)	2 (1.7%)	> 0.999
Potassium Citrate	0 (0.0%)	0 (0.0%)	
Other medications	17 (77.3%)	86 (74.1%)	0.757

^a^n (%)

^b^Pearson’s chi-square test; Fisher’s exact test

### Comparative analysis with cut-off point per test change

Analysis of the data with the cut-off point of the reference values for each laboratory test and 24-hour urine test, transforming the continuous variables into categorical ones, showed no association between electrolyte disorders and risk factors for the presence of kidney stones. Analysis of the 24-hour urine test also showed no difference between the groups studied (Table 7).

**Table 7:** Serum and urine laboratory tests

	Presence of kidney stones		
Features	No, *N* = 116^a^	Yes, *N* = 22^a^	p-value^b^
Total serum calcium			0.453
Normal	104 (89.7%)	19 (86.4%)	
High total calcium	11 (9.5%)	2 (9.1%)	
Reduced total calcium	1 (0.9%)	1 (4.5%)	
Serum ionic calcium			0.610
Normal	91 (83.5%)	17 (77.3%)	
High ionic calcium	17 (15.6%)	5 (22.7%)	
Reduced ionic calcium	1 (0.9%)	0 (0.0%)	
Serum sodium			0.183
Normal	103 (93.6%)	17 (85.0%)	
Hypernatremia	4 (3.6%)	1 (5.0%)	
Hyponatremia	3 (2.7%)	2 (10.0%)	
Serum potassium			0.647
Normal	103 (95.4%)	19 (95.0%)	
Hyperkalemia	3 (2.8%)	0 (0.0%)	
Hypokalemia	2 (1.9%)	1 (5.0%)	
Serum creatinine			0.088
Normal	90 (77.6%)	17 (77.3%)	
Hypercreatininemia	25 (21.6%)	3 (13.6%)	
Hypocreatininaemia	1 (0.9%)	2 (9.1%)	
Serum uric acid			0.162
Normal	57 (69.5%)	13 (81.2%)	
Hyperuricemia	24 (29.3%)	2 (12.5%)	
Hypouricemia	1 (1.2%)	1 (6.2%)	
Parathormone			0.814
Normal	56 (56.0%)	9 (52.9%)	
High	44 (44.0%)	8 (47.1%)	
Vitamin D			> 0.999
Reduced vitamin D	80 (85.1%)	13 (86.7%)	
Normal	14 (14.9%)	2 (13.3%)	
Calcium 24 h			0.539
Normal	97 (83.6%)	17 (77.3%)	
Hypercalciuria	19 (16.4%)	5 (22.7%)	
Sodium 24 h			0.147
Hypernatriuria	43 (53.8%)	5 (33.3%)	
Normal	37 (46.2%)	10 (66.7%)	
Potassium 24 h			0.059
Normal	67 (94.4%)	9 (75.0%)	
Reduced potassium	3 (4.2%)	3 (25.0%)	
High potassium	1 (1.4%)	0 (0.0%)	
Creatinine 24 h			0.309
Normal	90 (80.4%)	14 (70.0%)	
Reduced creatinine	20 (17.9%)	5 (25.0%)	
Elevated creatinine	2 (1.8%)	1 (5.0%)	
Citrate 24 h			0.766
Normal	61 (74.4%)	12 (70.6%)	
Hypocitraturia	21 (25.6%)	5 (29.4%)	
Oxalate 24 h			> 0.999
Normal	70 (94.6%)	14 (93.3%)	
Hyperoxaluria	4 (5.4%)	1 (6.7%)	
Uric acid 24 h			> 0.999
Normal	59 (83.1%)	11 (84.6%)	
High uric acid	12 (16.9%)	2 (15.4%)	
24 h urine volume			0.487
Urine volume below 2 L	82 (72.6%)	16 (80.0%)	
Normal	31 (27.4%)	4 (20.0%)	

^a^n (%)

^b^ Wilcoxon rank sum test; Pearson chi-square test; Fisher’s exact test

### Logistic regression

**Fig. 1:**
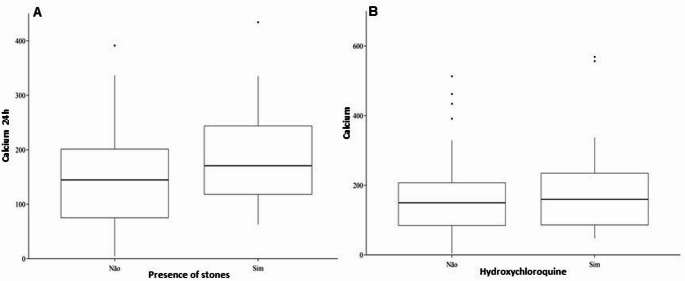
**A** *Bloxpot* Presence of calculus vs. 24-hour calciuria. **B** *Bloxpot* Use of hydroxychloroquine vs. 24-hour calciuria

In the logistic regression models, the variables analyzed did not show significant associations with the presence of kidney stones. In the first model, 24-hour calcium (p-value = 0.083), parathormone (p-value = 0.633), uric acid (p-value = 0.082), age (p-value = 0.657), and gender (p-value = 0.847) showed no significant association. In the second model, Hydroxychloroquine (p-value = 0.067) and 24 h Calcium (p-value = 0.182) were also not important, and the interaction between Hydroxychloroquine * 24 h Calcium showed no difference (p-value = 0.304) (Fig. 1A). In the third model, the Current clinical stage had no significant association (p-value = 0.299). In the fourth model, Hydroxychloroquine (p-value = 0.221), Adjusted Calciuria (p-value = 0.135), and the interaction Hydroxychloroquine * Adjusted Calciuria (p-value = 0.733) were not significant (Fig. 1B). The only variable with a potentially inverse association was uric acid but without statistical significance.

## Discussion

This study sought to fill the gap in knowledge about the urological manifestations of sarcoidosis [13], emphasizing the prevalence and risk factors related to the formation of kidney stones, as well as correlating the stage of the disease with urological findings. The patient’s demographic, metabolic, and radiological profile analysis aimed to identify predictors of severity and risk factors that may affect prognosis, providing essential data for more optimized disease management in urological clinical practice.

The most common symptoms in patients are dyspnea (74.6%) and cough (63.0%), related to lung involvement, which can lead to fibrosis in the advanced stages [3]. The disease also affects the skin, eyes, and cardiovascular system [14]. Skin involvement occurs in 80% of cases at diagnosis, essential for early identification [15]. Ocular involvement affects 10–50% of patients, with uveitis being the most common complication [16]. Cardiovascular involvement is generally asymptomatic but can include arrhythmias and atrioventricular blocks, causing chest pain, dyspnea, palpitations, and syncope [17]. Hypercalcemia and hypercalciuria increase the risk of kidney stones and are monitored in patients with sarcoidosis [18, 19], due to the extrarenal production of 1,25-dihydroxyvitamin D by sarcoid granulomas, leading to disturbances in calcium metabolism [20].

Specific follow-up to investigate kidney stones in patients with sarcoidosis is not routine. It can result in the underdiagnosis of nephrolithiasis and its complications, such as nephrocalcinosis and chronic kidney disease [7]. In our cohort, the prevalence of kidney stones was 15.9%, slightly higher than the rates reported in the general population (8.8% in the USA in 2012 and 10.6% in 2018; 7.5% in Europe) [21–23]. While this difference may not appear striking, it suggests that patients with sarcoidosis may be at increased risk, especially in the presence of persistent calcium metabolism disorders. However, as no sarcoidosis-specific factors were identified as predictors for stone formation, these results should be interpreted with caution. The prevalence of kidney stones in this follow-up is 3–14%, with a rate of 15.9%, higher than population studies [21]. Compared to data from the general population, the prevalence in the USA was 8.8% in 2012 and 10.6% in 2018, while in Europe it was 7.5% [22, 23]. These data highlight the importance of systematic screening for nephrolithiasis in sarcoidosis patients, especially those with persistent hypercalcemia. Patients with sarcoidosis also have comorbidities such as hypertension, diabetes, and dyslipidemia, which are associated with the formation of kidney stones [22]. Metabolic syndrome, characterized by chronic inflammation, insulin resistance, and systemic dysregulation, also contributes to this risk [24, 25]. The relationship between nephrolithiasis and hypertension is still uncertain, but endothelial dysfunction can favor the formation of crystals, and diabetes is associated with uric acid crystals [26].

When analyzing the profile of the patients, no specific subgroups or risk factors for stone formation were found. About demographic data, most of the patients surveyed were female (67.4%). The average age of the patients was 54 years, ranging from 47 to 60 years. These results are in line with previous studies that report a higher incidence of sarcoidosis in women, and the age range of 40 to 60 years is the most common time for diagnosing the disease [5]. In addition, the prevalence observed among women may suggest a genetic or hormonal predisposition that should be investigated in future studies. The literature indicates that sarcoidosis is more common in women, with a ratio of approximately 2:1 for men, especially in older age groups [5].

A history of previous renal lithiasis has been identified as a risk factor, with 30–50% of patients relapsing within 5–10 years, which reinforces the need for regular follow-up with imaging tests [27]. Regarding presentation and severity, most patients had moderate disease with a predominance of stage II (54.6%). In previous studies, the literature often reports a higher incidence of stage I cases, ranging from 45 to 60%. The discrepancy between our findings and the data from previous series may indicate a specific sample that has a more severe presentation of the disease due to their follow-up in a specialized outpatient clinic.

Analysis of the patient’s profile and 24-hour urine sought to exclude known factors that could modify metabolic aspects and impact stone formation, such as metabolic syndrome, uric acid disorders, parathyroidism, hypocitraturia, and hyperoxaluria, but these disorders were not found. The research focused on the impact of sarcoidosis on stone formation and alterations in calcium metabolism. Evaluation of the type of presentation and pulmonary classification (Scadding scale) also showed no association with stone formation or changes in hypercalciuria or hypercalcemia, showing no link with the pulmonary stage of the disease. Pulmonary changes do not indicate chronological evolution, but they do have prognostic value [28], with spontaneous resolution occurring mainly in stages 1 and 2, while persistence for more than 2 to 3 years suggests chronic evolution [29].

The clinical course of sarcoidosis is variable and can evolve from spontaneous resolution to chronic disease. The evolution can be limited, with two-thirds of cases being self-limiting in 12 to 36 months or chronic, with 10 to 30% of patients requiring continuous therapy [30]. Treatment is indicated for patients with vital organ involvement or functional risk, such as advanced pulmonary fibrosis, pulmonary hypertension, cardiac sarcoidosis, severe dermatological problems, posterior uveitis, and hypercalcemia [3]. Treatment aims to control the disease and metabolic changes. Cardiac, neurological, ocular, and renal involvement and hypercalcemia indicate treatment [27]. During follow-up, the patients were at different stages and were already receiving treatment when indicated. Ianuzzi observed hypercalciuria in 40% of patients and hypercalcemia in 11% [3]. Despite treatment, 9.4% had hypercalcemia, 16.8% had a change in ionic calcium, and 17.4% had hypercalciuria, with no positive association with the formation of kidney stones.

We observed a greater tendency to use hydroxychloroquine among stone-forming patients but with no statistically significant impact. Hydroxychloroquine, used in managing calcium disorders in sarcoidosis, modulates macrophage activity and vitamin D synthesis, which can regulate hypercalcemia and hypercalciuria, risk factors for kidney stones [31].

In this study, adjusted calciuria refers to 24-hour calciuria corrected for 24-hour creatinine to reduce bias from inadequate collections, such as abnormal urine volumes or incorrect collection times. This adjustment is mainly used to control pediatric patients [32]. Creatinine correction standardizes calcium excretion by the glomerular filtration rate, ensuring more accurate calcium measurement in the urine and considering variations in urinary dilution and concentration. This helps minimize the effects of inadequate collections and makes calciuria results more reliable and comparable.

Collecting a complete 24-hour urine sample, with analysis of all the necessary parameters, is a process that requires rigorous care, often using various preservatives and requiring between 2 and 3 days for collection. This logistical aspect was the main limitation of our study, resulting in the loss of many patients who could not complete the collection. Even in home collections, it is essential to ensure that the collection is correct for reliable results. Inadequate collections can falsely reduce or increase values. The ideal volume is 2000 ml/day for an adult, but in the study, 73.4% of patients had a volume below this value, with an average of 1650 ml/24 h. To reduce the impact, an analysis was carried out with creatinine-adjusted calciuria, resulting in an 18.3% increase in values without modifying the risk analysis. Hypercalciuria promotes renal alterations and stones, favoring nephrolithiasis and nephrocalcinosis in more advanced cases [18, 19]. However, therapeutic approaches with corticosteroids and ACE inhibitors can influence metabolic indicators, masking the fundamental frequency of hypercalciuria. Long-term follow-up could better demonstrate the impact of hypercalciuria, but case-control studies are not feasible due to the risk of metabolic and renal complications.

Macrophages activate vitamin D in the sarcoidosis granuloma in the lungs [3], and the dosage of 25-hydroxyvitamin D is low due to feedback and consumption mechanisms. In pre-treatment patients, hypercalciuria levels can reach 40% [3]. Many patients experience increased intestinal absorption of calcium and greater bioavailability. Patients with vitamin D deficiency (and normal ionized calcium levels in the blood) treated with ergocalciferol showed increased vitamin D storage and decreased levels of 1,25-dihydroxyvitamin D and angiotensin-converting enzyme, suggesting an effect on granulomatous inflammation [20]. However, some patients showed increased blood calcium, which requires further clinical studies to determine the safety and efficacy of vitamin D as a treatment. The changes in parathormone levels, with elevations observed in 44.4% of patients, contrast with the findings of early studies on sarcoidosis, which indicated suppressed parathormone levels due to pulmonary vitamin D activation [33]. Although rare cases of hyperparathyroidism associated with sarcoidosis have been reported, causality has not yet been established. This suggests the need for research exploring calcium metabolism and the interaction between different forms of vitamin D and parathormone in these patients. The high prevalence of hypovitaminosis D in Brazil and the population studied may partly explain the increased PTH levels observed in many of our sarcoidosis patients.

When monitoring patients, it is essential to consider the particularities of the disease and clinical follow-up. Due to its uncertain evolutionary nature [28, 34], sarcoidosis may have had different metabolic values at times before the start of treatment, which contributed to the formation of renal lithiasis. Studies evaluating pre-treatment conditions and the evolution of the disease are necessary to determine whether the risk groups, which are currently similar, were previously the same or whether the differences observed reflect the treatment.

A significant limitation of our study lies in the sample size and data loss, especially in the 24-hour urine collection. This loss could compromise the robustness of the conclusions since the analysis of metabolic parameters depends on the integrity of the data collected. Another limitation of our study is the limited availability of data on stone composition. We were able to retrieve composition results for four patients: three stones were composed of calcium oxalate and one of calcium phosphate. Although this small number does not allow definitive conclusions, it suggests a predominance of calcium-based stones, consistent with the pathophysiology of sarcoidosis-related hypercalciuria. Future studies including larger series with detailed stone analysis may reveal new associations between sarcoidosis and specific types of nephrolithiasis.

Despite these limitations, this study remains a pioneer in assessing nephrolithiasis in sarcoidosis in the Brazilian population, emphasizing calcium metabolism disorders as central factors and highlighting the importance of further studies that explore stone composition and long-term follow-up.

The majority of sarcoidosis patients are female, with an average age of 54, and the prevalence of nephrolithiasis is 16%, higher than in the general population. Calcium metabolism disorders, such as hypercalcemia and hypercalciuria, are present even during treatment, and it is essential to control them to avoid kidney stones. No significant associations were found between the stage of the disease and urological findings. To identify sarcoidosis in patients with nephrolithiasis, it is necessary to consider clinical, radiological, and metabolic aspects. A history of renal lithiasis should be considered, but no other clinical or laboratory factor was identified as an additional risk for differentiated follow-up.

## Data Availability

No datasets were generated or analysed during the current study.
